# Validation of a new smart textiles biotechnology for heart rate variability monitoring in sheep

**DOI:** 10.3389/fvets.2022.1018213

**Published:** 2022-11-22

**Authors:** Luca Turini, Francesca Bonelli, Antonio Lanatà, Valentina Vitale, Irene Nocera, Micaela Sgorbini, Marcello Mele

**Affiliations:** ^1^Dipartimento di Scienze Agrarie, Alimentari, Agro-ambientali, University of Pisa, Pisa, Italy; ^2^Centro di Ricerche Agro-Ambientali “E. Avanzi”, University of Pisa, Pisa, Italy; ^3^Dipartimento di Scienze Veterinarie, University of Pisa, Pisa, Italy; ^4^Dipartimento di Ingegneria dell'Informazione, University of Florence, Firenze, Italy

**Keywords:** sheep, ECG, heart rate variability (HRV), smart textile electrodes, validation

## Abstract

Robust Animal-Based Measures (ABMs) are fundamental in order to assess animal welfare, however in semi-extensive sheep farming systems is not easy to collect ABMs without inducing additional stress in the animals. Heart rate variability (HRV) is a non-invasive technique of assessing stress levels related to animal welfare. It is considered a sensitive indicator of the functional regulatory characteristics of the autonomic nervous system. Several studies investigated the use of HRV for welfare assessment in dairy cows while research on sheep is scarce. Moreover, assessing HRV in small ruminants at pasture is critical because of the lack of a solution adoptable for field conditions. This study aimed to evaluate if a smart textiles technology is comparable to a Standard base-apex electrocardiogram (ECG) for measuring HRV in small ruminants. Eight healthy Massese dairy sheep were recruited. Standard base-apex ECG and smart textiles technology (Smartex ECG) were simultaneously acquired for 5 min in the standing, unsedated, unclipped sheep. The ECG tracings were recorded when animals were standing quietly. The Bland-Altman test and the linear regression analysis were applied after parameter extraction in time, frequency, and non-linear methods to compare Smartex against standard base-apex ECG systems. The Bland-Altman test was applied to all HRV extracted parameters (Mean RR, pNN50, RMSSD, LF/HF, SampEn, SD1, SD2, stdRR) to evaluate the agreement between the two different instruments, and a linear regression analysis was performed to evaluate the relationship between the two methods. The smart textiles biotechnology was simple to wear and clean. It can be worn without using glue and without shaving the sheep's wool, limiting animal handling and stress. Bland Altman test reported a robust agreement between the two systems. In fact, the regression analysis of HRV parameters showed that half of the parameters recorded had an R2 coefficient >0.75. Results also showed a very small reproducibility coefficient that indicated that the two methods were really close to each other. Smartex textiles technology can be used for HRV evaluation in sheep species as a potential ABM for animal welfare assessment.

## Introduction

In December 2020, Italy's sheep sector had a population of 7,034.16 thousand; 93.5 percent of the sheep were grown in marginal regions that were prone to desertion (1). In Italy, most sheep farming systems may be classified as “semi-intensive” or “semi-extensive”, (2, 3). Animals in a semi-intensive farming system are confined at night and when adverse weather conditions occur. Sheep are frequently kept on fenced or unfenced pastures that are either owned or rented. Animals in semi-extensive farming systems are always kept on pasture, and temporary confinement is used only when weather conditions are adverse (4). Evaluating the health and welfare of sheep is important in both farming methods, but it may be challenging, especially at pasture, where no formal evaluation technique has been defined (5). COST Action 846 on “Measuring and Monitoring Welfare” was formed in 2001 by the European “measuring welfare” working group with the objective of encouraging and improving research on stress and welfare status in farm animals (6). The Action project team brought together professionals in animal and veterinary sciences, assisting in the identification of heart rate variability (HRV) as a non-invasive method of measuring stress levels in animals (7, 8). HRV, defined as the fluctuations in the heart rhythm or rate, is regarded as a sensitive measure of the autonomic nervous system's functional regulation features (6, 8). Different methods have been used to assess animal welfare in all the species based on autonomic physiologic condition; in pigs, the HRV measured by electrocardiogram (ECG) was used for monitoring the parasympathetic tone activity (PTA) under different anesthetic conditions (9, 10), in horses the same method for HRV evaluation was used for assessing whether the PTA index was able to predict changes in mean arterial pressure in horses anesthetized with different anesthetic protocols and with different health conditions (11, 12). Moreover, HRV evaluated by ECG was used along with heart rate and cortisol levels to evaluate different responses to training in horses (13). HRV has been evaluated in bovines for monitoring circadian rhythm, seasonal influences, sickness, and welfare (14–23). Finally, in sheep HRV has been investigated especially in experimental conditions in fetuses for assessing fetal viability, prematurity, and acidosis (24–27). Due to the lesser economic importance of sheep production, there has been little investigation into HRV in sheep for welfare assessment.

Several human-developed bioengineering solutions for ECG evaluation, have been already adapted to domestic animals (28–32). Smart textiles are considered a very promising technology due to easiness in the use. Smart textiles, in fact, may be placed without the need for sticky electrodes, glue, or cohesive bandages, and they can capture a highly consistent signal, resulting in better HRV monitoring (33, 34). Due to the challenging nature of measuring HRV in small ruminants on pasture, the present preliminary study, aiming to examine if a smart textile technology could be compared to a Standard base-apex ECG for measuring HRV in small ruminants, was set in farm conditions. Our study hypothesis was that the two systems were similar, and that smart textile technology could be used in field conditions.

## Materials and methods

This prospective observational study was approved by the Institutional Animal Care and Use Committee of the University of Pisa (49/2019). An owner's written consent was obtained for ECG recording for the sheep included in the study.

### Animals and management

Eight healthy adult female Massese sheep (mean age 8.6 ± 1.5 years and weight 40.8 ± 2.1 kg) were recruited from the Centro di Ricerche Agro-Ambientali “E. Avanzi,” of the University of Pisa. Massese sheep present an open or semi-open fleece, with conical tufts with rather smooth short wool (50–75 mm), lead-gray in color, and with a lighter-colored apical part in females which is almost black in the males. During the day, the animals were housed in a grazing area (0.5 ha), and at night, they were kept in a separate barn. Sheep in both places had unlimited access to hay and clean water. Only non-lactating and non-pregnant sheep were included in the study. Furthermore, sheep had to be healthy at the time of the study based on their history and on physical and clinical examination (35). Sheep were excluded from the study if they were affected by cardiovascular or other diseases during the experimental period.

### Smartex system information

**Figure 1:**
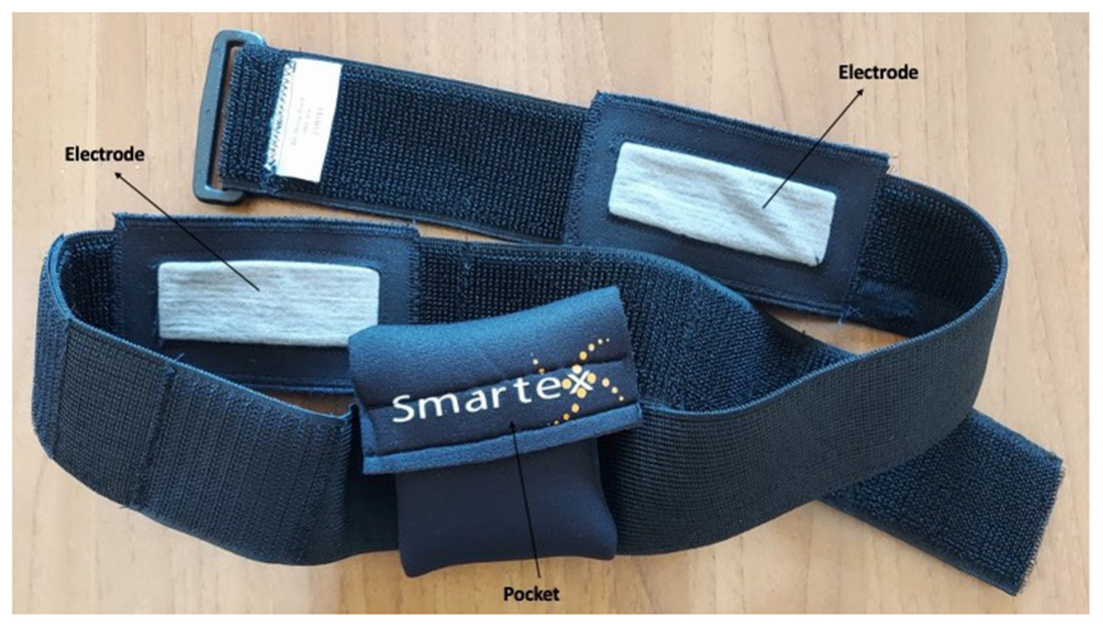
Smartex system. Smartex system composed of an elastic band with a couple of electrodes and with a neoprene pocket.

A new wearable system has been designed to monitor sheep in their natural environment. The system is based on the use of textile fabric electrodes, that can be applied for prolonged time periods as the fabric allows a reliable contact between the electrode and the skin of the animal. The results gained from this physical contact are more efficient compared with standard electrodes that are coupled by gel, like the Ag/AgCl ones (i.e., electrode composites of silver and silver salt). When Ag/AgCl electrodes are used a coupling gel is inserted in the interface between the electrode and skin to adapt the electrical impedance and keep a high level of humidity and good signal level consequently. However, the hydration level of gel decreases with time, while the humidity of a textile electrode, specially designed according to a multi-layered to capture the water, can persist for a longer period. Moreover, the structure of the fabric makes the electrode flexible and conformable to the skin. Within this study, Smartex realized the first dedicated prototype, conceived as a tool to evaluate the most functional position of the electrodes on the sheep. The prototype combines an elastic band, realized according to the mean size of the sheep, with a couple of electrodes that can be moved in different positions, on the inner side of the band, for having the best electrode locations on the animal's skin. These electrodes are connected through a flexible textile-compatible wire with a connector that can be plugged into a portable electronic device (RUSA, *dimensions:* 52 X 50 X 15 mm, *weight:* 50 gr). The electronic device has been protected with a neoprene pocket to survive the harsh environment, as the system has been conceived to be used in a wild natural context. After the preliminary evaluation of the position of the electrodes, a new system has been realized with the electrodes fixed on the belt in a position corresponding to the belly of the sheep, and the wires have been integrated into the belt to avoid any risks of getting tangled in bushes during pasture, which can easily generate the rupture of the electrical contact (Figure 1). The portable electronic devices, placed on the back of the sheep, were able to acquire one standard ECG lead at 250 Hz and were also equipped with an Inertial Motion Unit (IMUs) to monitor the sheep's physical activity, which was acquired at a sampling frequency of 25 Hz. The device was battery powered and it was able to collect data continuously for 24 h. In addition, the device was wirelessly connected *via* Bluetooth to a mobile device to check the status of the signals and remotely control the storing process on a digital card of the acquired physiological data.

### Data acquisition

**Figure 2:**
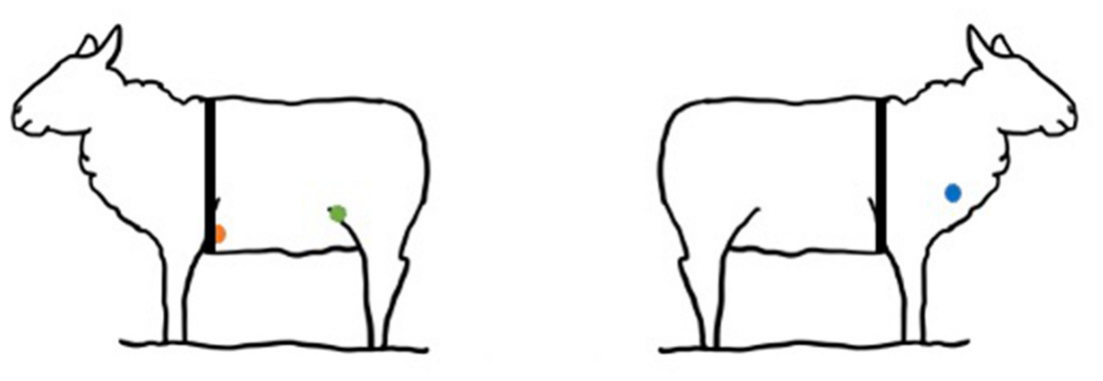
Placement of Smartex and Televet electrodes. Televet: the positive electrode (orange circle) was placed at the cardiac apex at the 5^th^ left intercostal space level, and the negative electrode (blue circle) was placed on the jugular furrow in the bottom third of the left side of the neck. The 3rd electrode (green circle) was placed away from the heart on-site. Smartex: the elastic band with the textile electrodes (rectangular, 6.5 × 2.5 cm) was positioned vertically no more than 2 cm below the positive ECG electrode.

The ECGs were acquired in 1 day at the farm between the hours of 9.00 and 11:00 a.m. The sheep were restrained in a box for 10 min before the ECG was recorded. To minimize stress reactions in sheep isolated from the rest of the flock, the restraint box was put inside the herd. The investigation was carried out with the help of the shepherd. Standard base-apex ECG and Smartex ECG (hereinafter called Smartex) were simultaneously acquired for 5 min in the standing, non-sedated, unclipped sheep. Time recording was decided because previous studies showed that analyzing 5-min segments (short term) or < 5-min segments (ultra-short term) of inter-beats intervals in healthy individuals' data produce results comparable to, or even better than, analyzing 24 h of data (36–39). A small amount of 70% isopropyl alcohol was applied directly before each session and was used to optimize contact and obtain a good-quality ECG signal for both methods. The ECG tracings were taken while the animals stood still. As previously described (31, 40), a conventional base-apex ECG (TeleVet 100, Kruuse, Denmark) was obtained (hereinafter called Televet). Animals' ECGs were measured using crocodile clamp electrodes (CCE) and sticky gel. The positive CCE (left arm) was placed at the cardiac apex at the 5th left intercostal space level, and the negative CCE (right arm) was placed on the jugular furrow in the bottom third of the left side of the neck. The 3rd CCE (ground lead) was placed away from the heart on-site. Two smart textile electrodes were attached to a portable data logger in the Smartex ECG (Smartex Srl, Navacchio, Italy). The textile electrodes (rectangular, 6.5 × 2.5 cm) were positioned vertically no more than 2 cm below the standard ECG electrodes (Figure 2). A single lead ECG trace (lead II) was obtained by each ECG recording device (Televet and Smartex). The single lead has been acquired throughout the use of the two electrodes as the voltage difference between electrodes. Electrodes were kept in place using an elastic belt fastened around the chest behind the sheep's shoulder.

### Data processing

The first stage of data processing was to preprocess ECG signals for further analysis. In the preprocessing stage, ECG signals were segmented into 1-min time laps; the first-time lap was excluded from analysis and used for synchronizing the two monitoring ECG systems. As a matter of fact, 4-time laps were used for analyzing data for each animal. In each time lap, HRV was extracted from ECG data, and numerous parameters were computed from it to characterize cardiovascular dynamics (the parameters are reported in the HRV parameters extraction section). The analysis was carried out in time-domain, frequency-domain, and non-linear indices. Nonlinear parameters were extracted using the latter technique. Data from the heart rate monitor was imported into Kubios^®^ HRV Standard [Kubios HRV software (version 3.0.2), Biomedical Signal Analysis Group, Department of Applied Physics, University of Kuopio, Finland] for analysis of time-domain, frequency-domain, and non-linear indices to determine HRV measures. A medium artifact correction (automatic beat correction algorithm) was used to decrease inaccuracy throughout the sampled ensemble as a first step in creating consistency in signal analysis (41–43). These data were not subjected to detrending. All HRV analyses from Kubios were reviewed for the percentage of artifacts corrected. Kubios' results from extracted HRV can be found in the results section.

### HRV parameters extraction

After the identification of the R-peak, where R is a point corresponding to the peak of the QRS complex of the ECG wave, we computed the time series composed from the time intervals between consecutive R peaks (i.e., RR time series). HRV was the variation in the time interval between heartbeats, and it was measured by the variation in the beat-to-beat interval. In this study, HRV variability parameters were extracted in the time, frequency, and non-linear domains. Specifically, in the time domain, we extracted the mean value of the RR time series (meanRR), the standard deviation of the RR time series (stdRR), the consecutive RR intervals minor of fifty milliseconds (pNN50), the root mean square of successive differences between normal heartbeats (RMSSD). In the frequency domain, we computed the ratio between the low-frequency band (LF), from 0.04 to 0.15 Hz, and the high-frequency band (HF), from 0.15 to 0.4 Hz (6, 44). LF/HF was considered an index of sympathovagal balance since the powers at LF and HF bands are assumed to be the result of sympathetic and parasympathetic modulation to the heart, respectively. In the non-linear domain, we extracted the sample entropy (SampEn), which is used for assessing the complexity of physiological time-series signals. Finally, the standard deviation of instantaneous beat-to-beat interval variability (SD1) and the continuous long-term R/R interval variability (SD2) from the Poincaré scatter plot of RR(n) vs. RR(n+1) were extracted. Where RR(n) was the time between two successive R peaks and RR(n+1) was the time between the next two successive R peaks when the ellipse-fitting technique adjusts the plot.

### Data analysis

After parameters extraction, many tests in time, frequency, and non-linear methods were used to compare Smartex to Televet. The distribution of the data was evaluated by the Kolmogorov–Smirnov test and the data were expressed as median ± mean absolute deviation (MAD). The Bland-Altman test was used to determine the agreement between the data collected from the two different instruments. It is usual to compute 95% limits of agreement for each comparison (average difference ± 1.96 standard deviations), in order to evaluate how far apart two techniques' measurements were more likely to be for the majority of the examination period. In the present study, the analysis evaluated the bias and the 95% limits of agreement between Televet vs. Smartex ECGs for all the parameters extracted (Mean RR, pNN50, RMSSD, LF/HF, SampEn, SD1, SD2, stdRR). Moreover, being the two-system affected by the same kind of errors the linear regression model II for non-parametric data has been applied. Furthermore, for taking into consideration the quality of the agreement for repeated measures with two different systems the coefficient of reproducibility has been computed (45).

Bland-Altman test and linear regression analysis were made using the commercial software MatLab (MathWorks^®^, Natick, MA, USA) to compute any significant difference among the parameters. Statistical analysis which takes into account repeated measurements was applied. A *P* < 0.05 was considered significant.

## Result

Table 1 reports median ± mean absolute deviation (MAD) of the parameters extracted with Smartex and Televet systems.

**Table 1:** Median value ± mean absolute deviation (MAD) of the parameters extracted.

**Parameter**	**Smartex**	**Televet**
Mean HR (bpm)	95.796 ± 9.496	96.299 ± 11.023
Mean RR (ms)	634.7 ± 66.7	632.9 ± 64.8
pNN50 (%)	38.880 ± 23.116	40.84 ± 24.346
RMSSD (ms)	81.5 ± 51.7	95.2 ± 56.4
LF/HF	0.277 ± 0.403	0.202 ± 0.334
SampEn	1.276 ± 0.405	1.287 ± 0.380
SD1	0.058 ± 0.037	0.068 ± 0.040
SD2	0.078 ± 0.028	0.081 ± 0.030
stdRR (ms)	80.1 ± 29.0	79.3 ± 31.9

Mean RR, mean value of the RR time series; pNN50, consecutive RR intervals minor of fifty milliseconds; RMSSD, root mean square of successive differences between normal heartbeats; LF/HF, index of sympathovagal balance; SampEn, sample entropy; SD1, standard deviation of instantaneous beat-to-beat interval variability; SD2, continuous long-term R/R interval variability; stdRR, standard deviation of RR.

**Figure 3:**
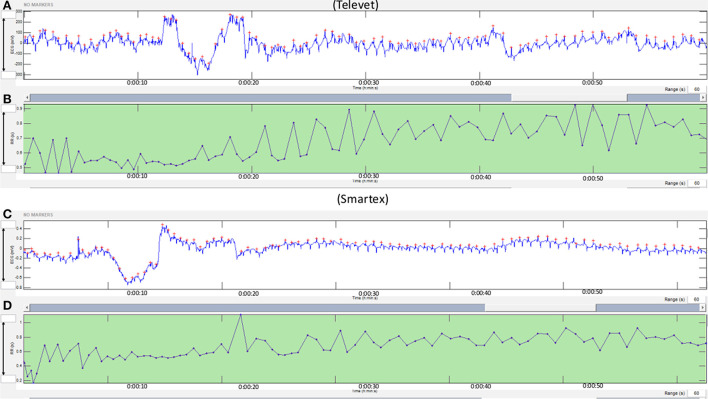
Synchronous acquisition for Smartex and Televet systems. The red circle in the figure highlights the absence or presence of movement artifacts in ECG signals. The figure shows a movement artifact in the Televet system and not in the Smartex. This signal noise can be responsible for the erroneous detection of R peaks from Kubios software. **(A)**: Televet ECG expressed in millivolt; **(B)**: Televet RR distance expressed in second; **(C)**: Smartex ECG expressed in millivolt; **(D)**: Smartex RR distance expressed in second. Total acquisition for both methods = 60 seconds.

Figure 3 shows a synchronous acquisition for Smartex and Televet systems. In both cases, no missed heartbeat occurred.

The Bland Altman plots are reported in Figures 4–11. In the details, a general agreement between the two systems was confirmed. The reproducibility coefficient (RPCnp) was calculated, and it showed a low value, meaning a great similarity between the two systems.

**Figure 4:**
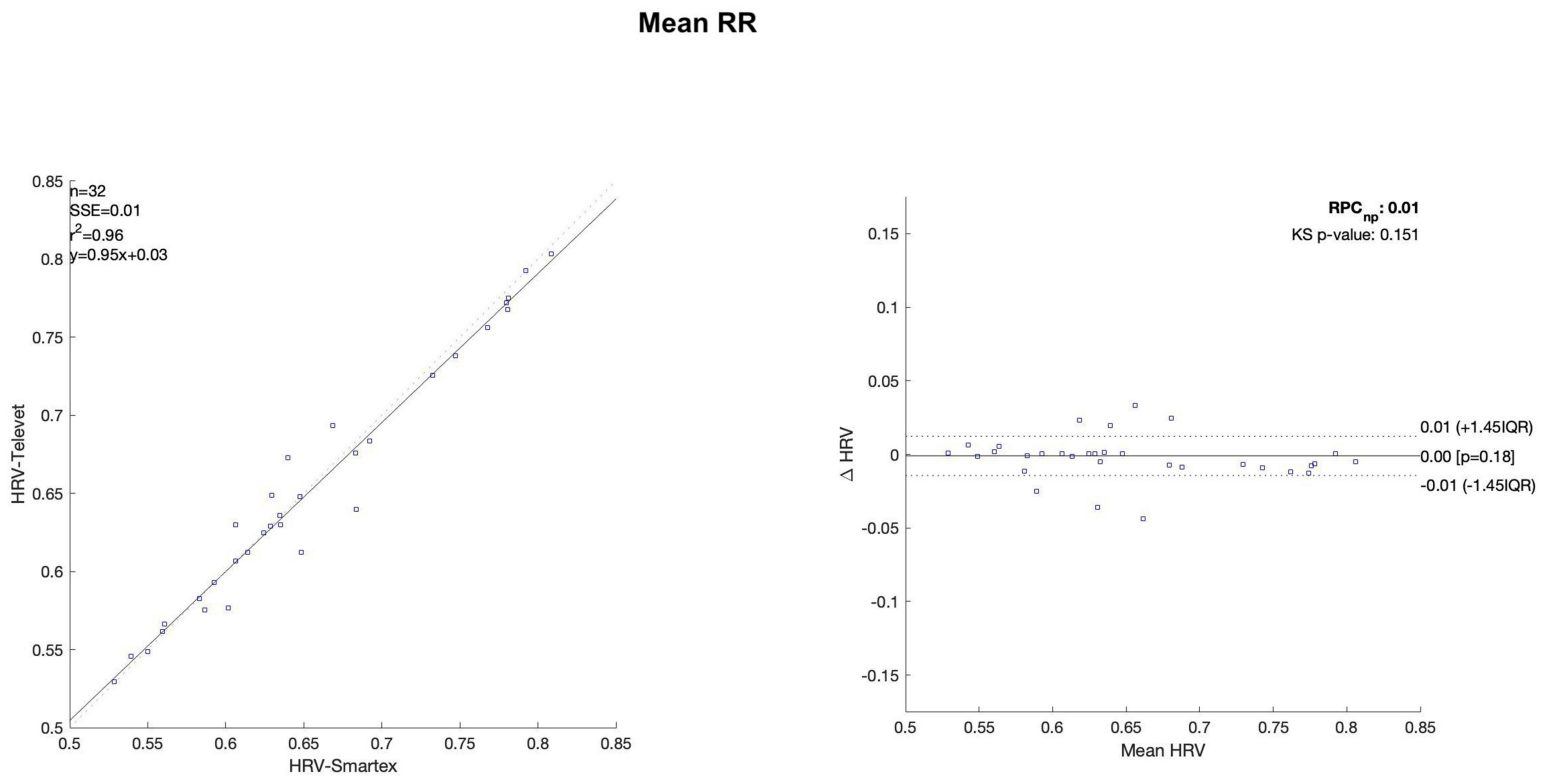
Bland Altman plots and the regression analysis results to compare Smartex *vs*. Televet. The **(Left)** part of the figure shows the regression analysis [best-fit line, number of elements (*n*), the squared Pearson correlation coefficient (r^2^), the sum of square error (SSE), and the linear equation (*y*)], in the figure, the boxes are paired measures, the line is the correlation. The **(Right)** part of the figure shows the Bland Altman plot with the limits of agreement [LOA, as dotted lines along with Interquartile Range (IQR)], the Kolmogorov Smirnov (KS) test results, and the reproducibility coefficient (RPCnp, which is expressed as percentage value estimated through IQR). Mean RR–mean value of the RR time series.

**Figure 5:**
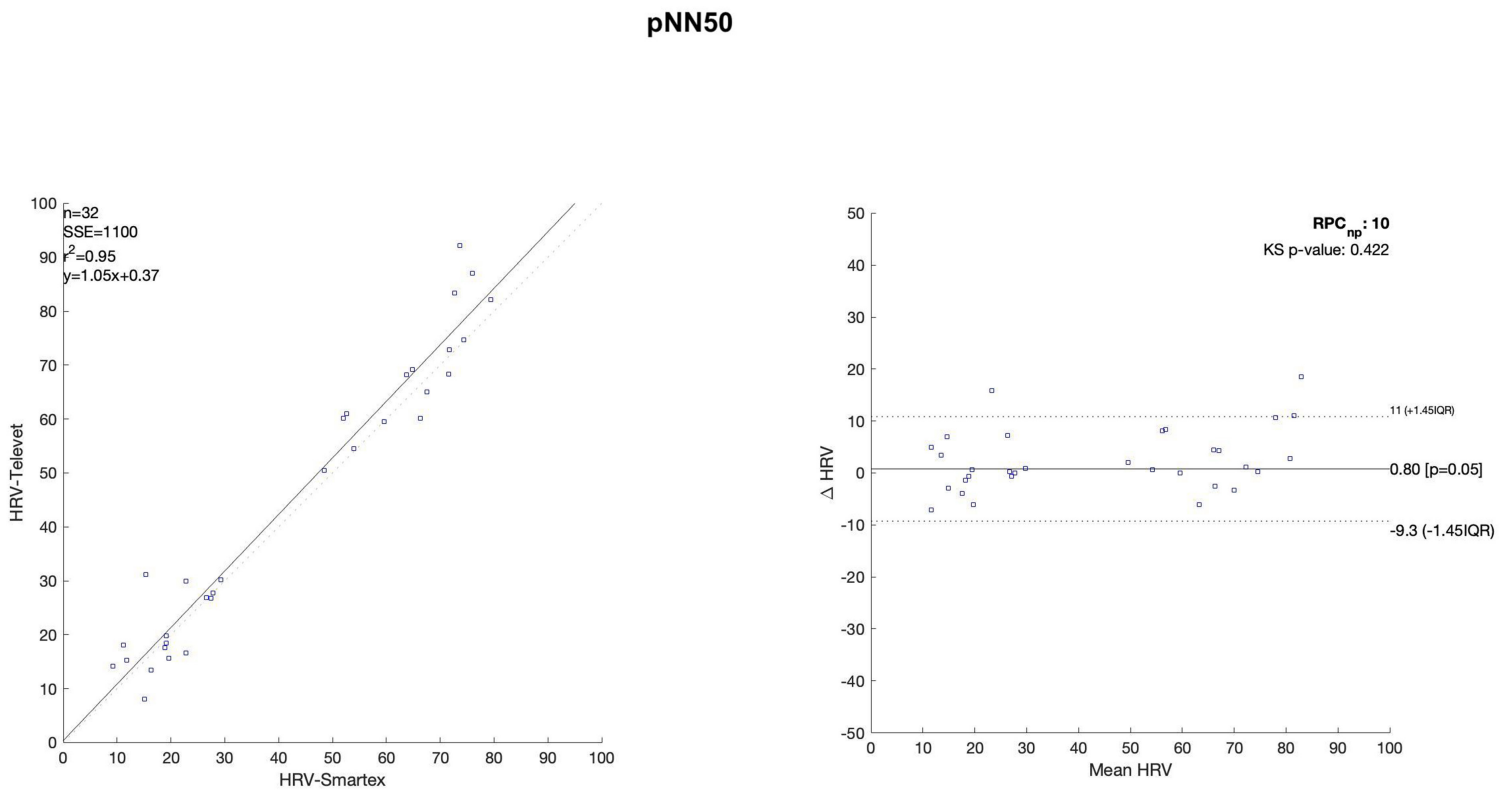
Bland Altman plots and the regression analysis results to compare Smartex *vs*. Televet. The **(Left)** part of the figure shows the regression analysis [best-fit line, number of elements (*n*), the squared Pearson correlation coefficient (r^2^), the sum of square error (SSE), and the linear equation (*y*)], in the figure, the boxes are paired measures, the line is the correlation. The **(Right)** part of the figure shows the Bland Altman plot with the limits of agreement [LOA, as dotted lines along with Interquartile Range (IQR)], the Kolmogorov Smirnov (KS) test results, and the reproducibility coefficient (RPCnp, which is expressed as percentage value estimated through IQR). pNN50–consecutive RR intervals minor of fifty milliseconds.

**Figure 6:**
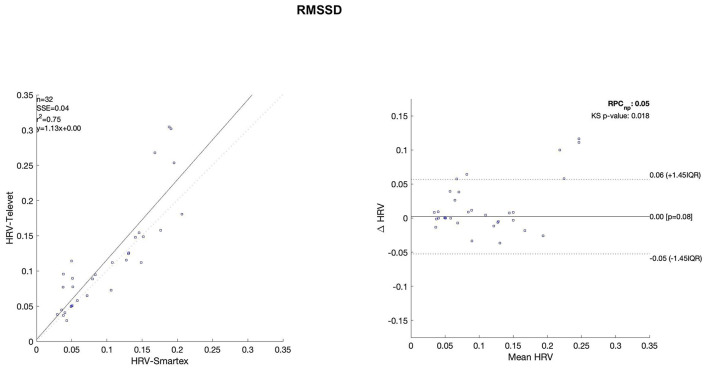
Bland Altman plots and the regression analysis results to compare Smartex *vs*. Televet. The **(Left)** part of the figure shows the regression analysis [best-fit line, number of elements (*n*), the squared Pearson correlation coefficient (r^2^), the sum of square error (SSE), and the linear equation (*y*)], in the figure, the boxes are paired measures, the line is the correlation. The **(Right)** part of the figure shows the Bland Altman plot with the limits of agreement [LOA, as dotted lines along with Interquartile Range (IQR)], the Kolmogorov Smirnov (KS) test results, and the reproducibility coefficient (RPCnp, which is expressed as percentage value estimated through IQR). RMSSD–root mean square of successive differences between normal heartbeats.

**Figure 7:**
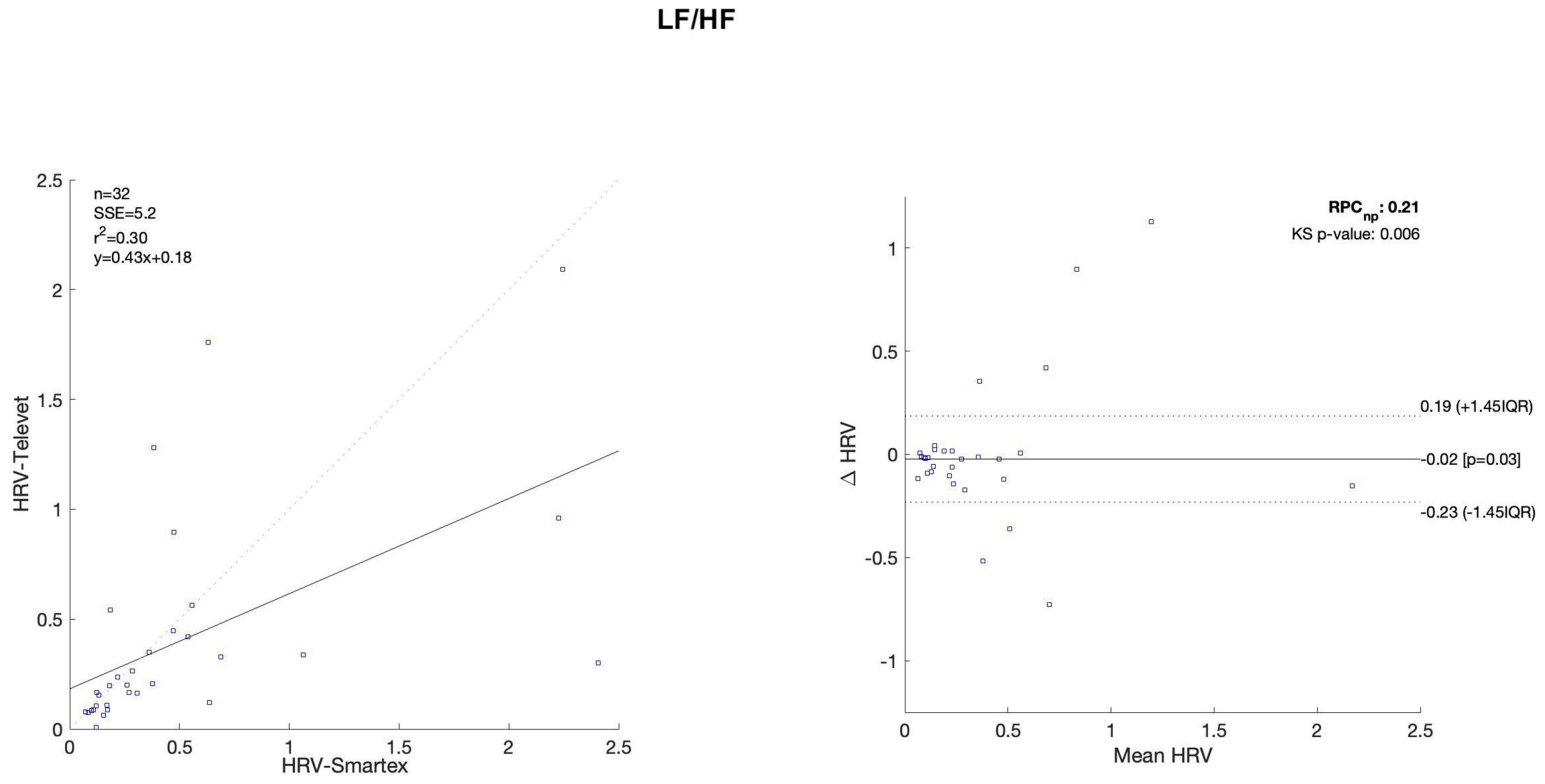
Bland Altman plots and the regression analysis results to compare Smartex *vs*. Televet. The **(Left)** part of the figure shows the regression analysis [best-fit line, number of elements (*n*), the squared Pearson correlation coefficient (r^2^), the sum of square error (SSE), and the linear equation (*y*)], in the figure, the boxes are paired measures, the line is the correlation. The **(Right)** part of the figure shows the Bland Altman plot with the limits of agreement [LOA, as dotted lines along with Interquartile Range (IQR)], the Kolmogorov Smirnov (KS) test results, and the reproducibility coefficient (RPCnp, which is expressed as percentage value estimated through IQR). LF/HF–index of sympathovagal balance.

**Figure 8:**
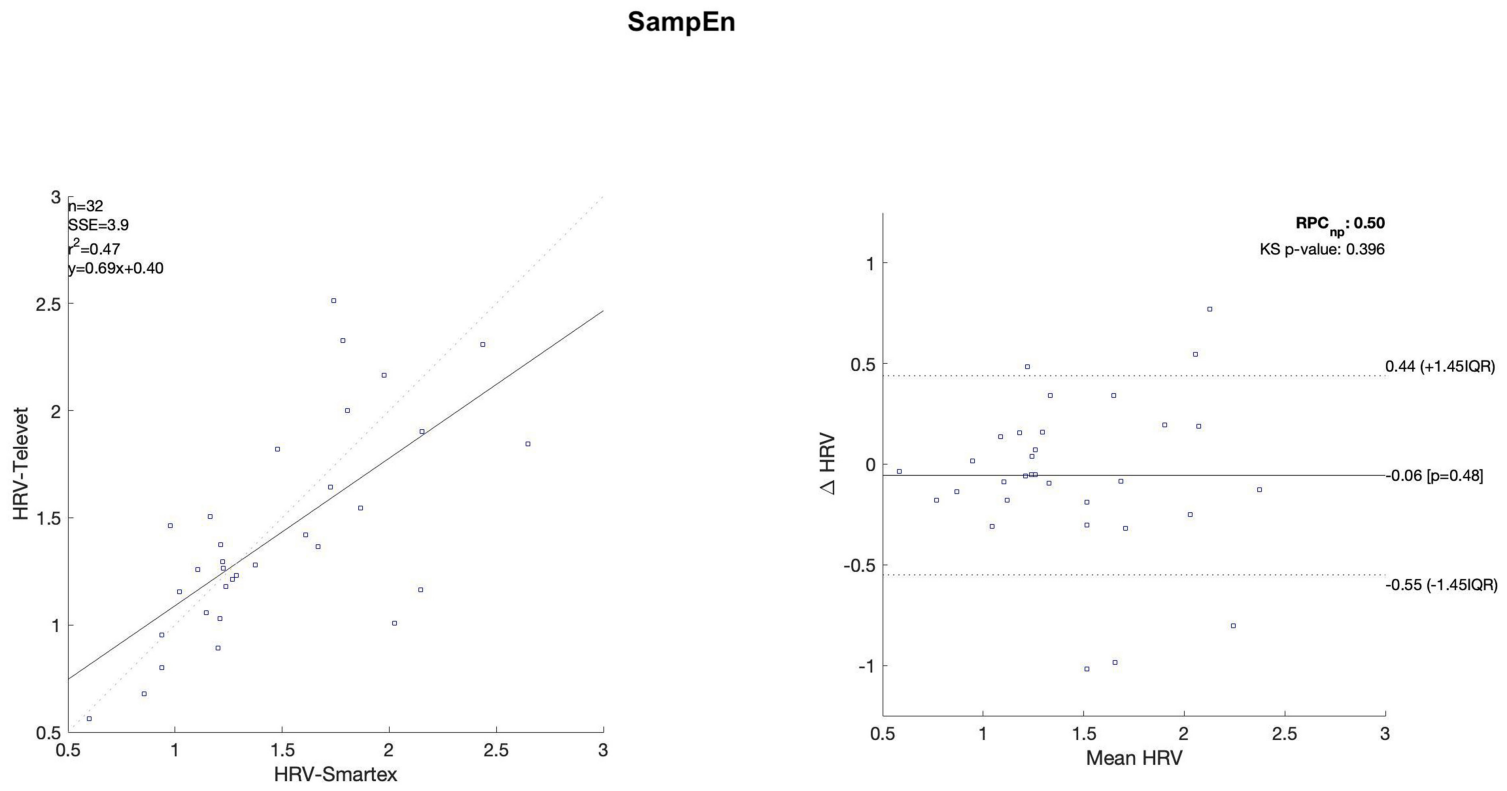
Bland Altman plots and the regression analysis results to compare Smartex *vs*. Televet. The **(Left)** part of the figure shows the regression analysis [best-fit line, number of elements (*n*), the squared Pearson correlation coefficient (r^2^), the sum of square error (SSE), and the linear equation (*y*)], in the figure, the boxes are paired measures, the line is the correlation. The **(Right)** part of the figure shows the Bland Altman plot with the limits of agreement [LOA, as dotted lines along with Interquartile Range (IQR)], the Kolmogorov Smirnov (KS) test results, and the reproducibility coefficient (RPCnp, which is expressed as percentage value estimated through IQR). SampEn–sample entropy.

**Figure 9:**
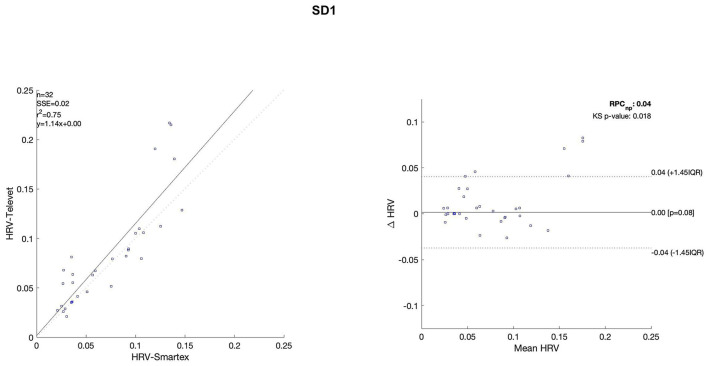
Bland Altman plots and the regression analysis results to compare Smartex *vs*. Televet. The **(Left)** part of the figure shows the regression analysis [best fit line, number of elements (*n*), the squared Pearson correlation coefficient (r^2^), the sum of square error (SSE), and the linear equation (*y*)], in the figure, the boxes are paired measures, the line is the correlation. The **(Right)** part of the figure shows the Bland Altman plot with the limits of agreement [LOA, as dotted lines along with Interquartile Range (IQR)], the Kolmogorov Smirnov (KS) test results, and the reproducibility coefficient (RPCnp, which is expressed as percentage value estimated through IQR). SD1–standard deviation of instantaneous beat-to-beat interval variability.

**Figure 10:**
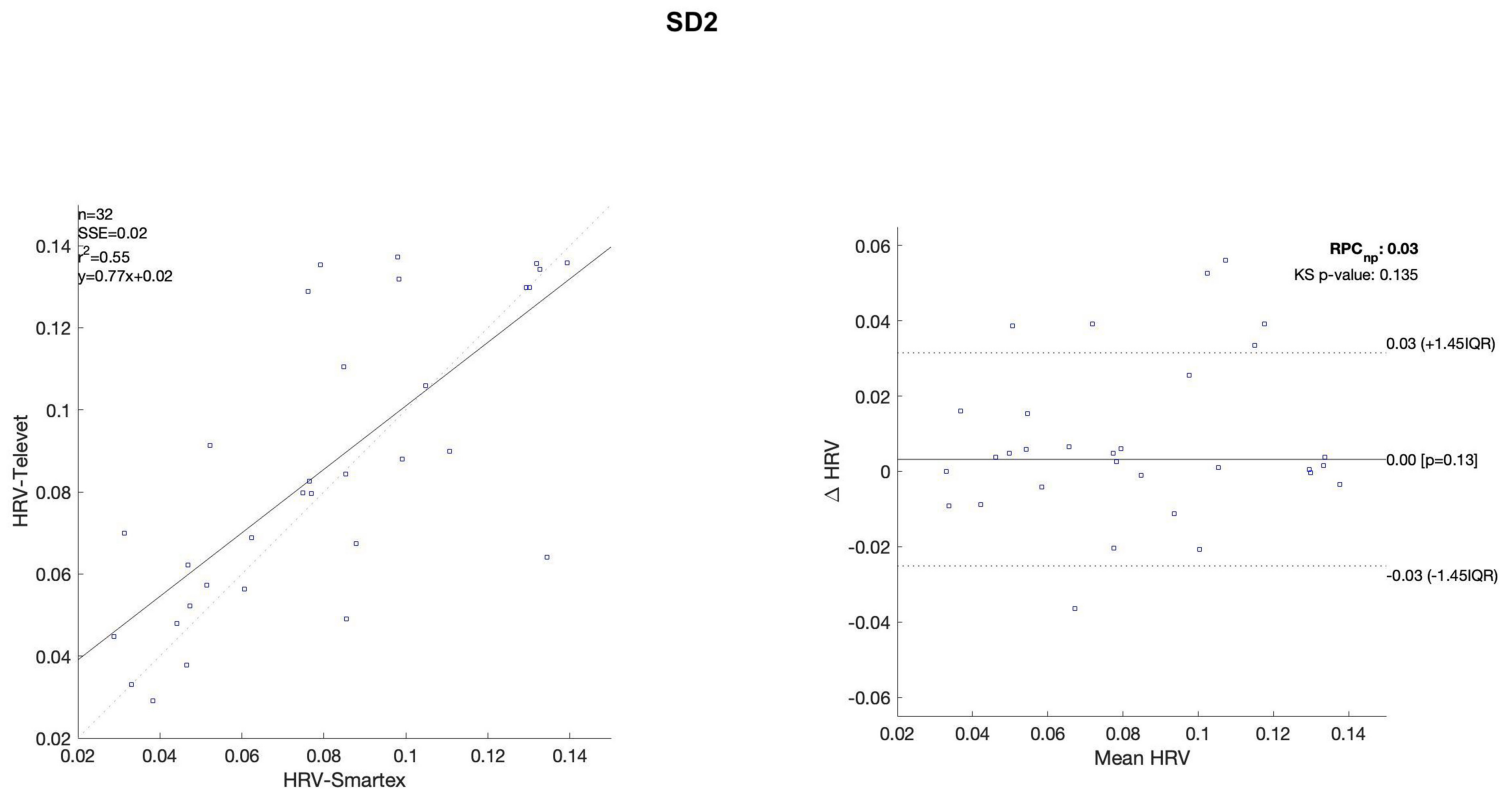
Bland Altman plots and the regression analysis results to compare Smartex *vs*. Televet. The **(Left)** part of the figure shows the regression analysis [best-fit line, number of elements (*n*), the squared Pearson correlation coefficient (r^2^), the sum of square error (SSE), and the linear equation (*y*)], in the figure, the boxes are paired measures, the line is the correlation. The **(Right)** part of the figure shows the Bland Altman plot with the limits of agreement [LOA, as dotted lines along with Interquartile Range (IQR)], the Kolmogorov Smirnov (KS) test results, and the reproducibility coefficient (RPCnp, which is expressed as percentage value estimated through IQR). SD2–continuous long-term R/R interval variability.

**Figure 11:**
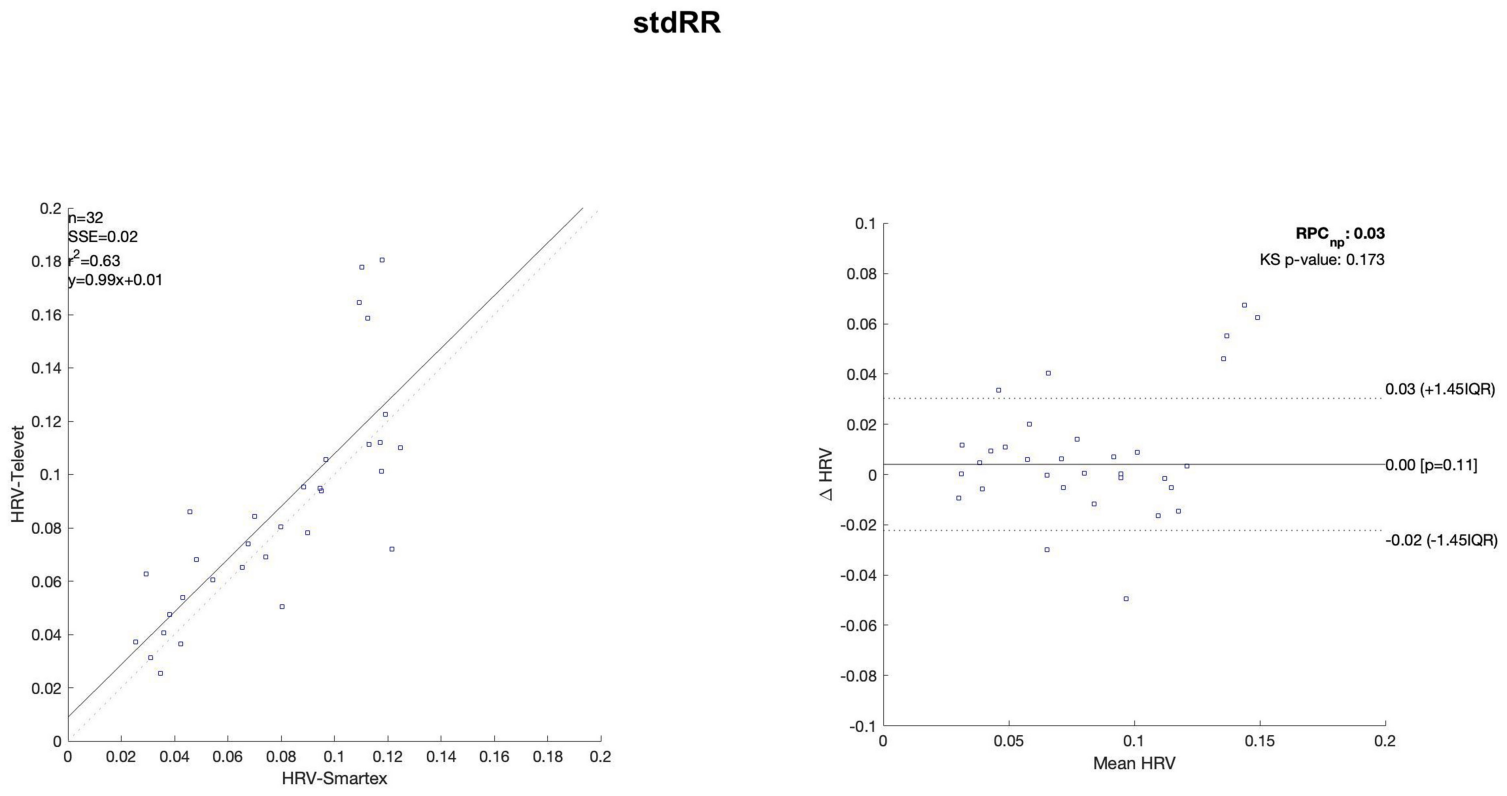
Bland Altman plots and the regression analysis results to compare Smartex *vs*. Televet. The **(Left)** part of the figure shows the regression analysis [best-fit line, number of elements (*n*), the squared Pearson correlation coefficient (r^2^), the sum of square error (SSE), and the linear equation (*y*)], in the figure, the boxes are paired measures, the line is the correlation. The **(Right)** part of the figure shows the Bland Altman plot with the limits of agreement [LOA, as dotted lines along with Interquartile Range (IQR)], the Kolmogorov Smirnov (KS) test results, and the reproducibility coefficient (RPCnp, which is expressed as percentage value estimated through IQR). stdRR–standard deviation of RR.

Table 2 showed the linear regression analysis of the extracted parameters. The regression analysis showed that four out of eight parameters have an R^2^ coefficient >0.75, highlighting a robust relationship between the two systems. Two parameters (LF/HF and SampEn) showed an R^2^ coefficient smaller than 0.5 and the remaining two parameters have an R2 coefficient >0.55 and smaller than 0.75. In Table 3 the values of the Bland-Altman concordance test are evaluated between the two methods.

**Table 2:** Regression analysis of the extracted parameters.

**Parameter**	**Equation**	**R^2^**
Mean RR	Y= 0.95x + 0.03	0.96
pNN50	Y= 1.05x + 0.37	0.95
RMSSD	Y= 1.13x + 0.00	0.75
LF/HF	Y= 0.43x + 0.18	0.30
SampEn	Y= 0.69x + 0.4	0.47
SD1	Y= 1.14x + 0.00	0.75
SD2	Y= 0.77x + 0.02	0.55
stdRR	Y= 0.99x + 0.01	0.63

Mean RR, mean value of the RR time series; pNN50, consecutive RR intervals minor of fifty milliseconds; RMSSD, root mean square of successive differences between normal heartbeats; LF/HF, index of sympathovagal balance; SampEn, sample entropy; SD1, standard deviation of instantaneous beat-to-beat interval variability; SD2, continuous long-term R/R interval variability; stdRR, standard deviation of RR.

**Table 3:** Values of Bland-Altman concordance test.

	**Mean RR**	**pNN50**	**RMSSD**	**LF/HF**	**SampEn**	**SD1**	**SD2**	**stdRR**
N°	32	32	32	32	32	32	32	32
ρ	0.97	0.94	0.88	0.79	0.68	0.88	0.73	0.84
SSE	0.007	0.0001	0.04	5.24	3.86	0.02	0.02	0.02
RMSE	0.001	6.03	0.04	0.41	0.36	0.03	0.02	0.02
slope	0.95	1.05	1.13	0.43	0.69	1.13	0.77	0.99
CV	2.33	13.83	36.22	120.02	26.64	36.23	30.08	30.70
rpcNP	0.01	10.06	0.05	0.21	0.49	0.04	0.03	0.03

ρ, Pearson correlation; SSE, sum square error, RMSE, root mean square error, slope, steepness of a line; CV, variation coefficient; rpcNP, reproducibility coefficient. Mean RR, mean value of the RR time series; pNN50, consecutive RR intervals minor of fifty milliseconds; RMSSD, root mean square of successive differences between normal heartbeats; LF/HF, index of sympathovagal balance; SampEn, sample entropy; SD1, standard deviation of instantaneous beat-to-beat interval variability; SD2, continuous long-term R/R interval variability; stdRR, standard deviation of RR.

## Discussion

Several studies showed the practical implication of evaluating the HRV for welfare assessment in dairy cows (14–21), while literature about small ruminants only reported HRV evaluation performed in experimental animals (lamb fetus and pregnant ewes) (24, 46, 47). Our study used textile electrodes as an alternative method to evaluate ECG–and the relative HRV–in sheep. HRV was characterized using the time and frequency domain to evaluate the correlation between the two different systems to evaluate ECG in sheep.

Smartex and Televet devices were both worn at the same time but Smartex seemed to be more comfortable, worn without any encumbrance or wired connection, making the animal free to move, moreover, the recording procedure was faster. In addition, one of the big advanced of using this system is the absence of an electrolytic gel layer between electrodes and the animal skin/wool, due to the good level of electrical coupling and the adaptability of the textile band to the animal skin morphology.

The literature described HRV as 24-h, short-term (~5 min) or brief, and ultrashort-term (< 5 min). We decided to evaluate a time period of 4 min, thus using an ultrashort-term interval, because it was stated that it can be used for healthy individuals, being superior to analyzing 24 h of data (39).

Concerning the biometric analysis of HRV parameters, it was possible to compare results from our study with data mainly obtained by ECG and Polar^®^ technology in different species (17, 22, 48–50). The mean HR parameter from sheep in our study was slightly lower compared to dogs (50), and slightly higher compared to horses (49) and cows (17) reflecting the physiological differences between these species. The mean RR obtained in sheep from the present study by using both technologies was similar compared to pigs and dogs (48, 50), but slightly lower compared to horses (49) and cows (17), while the stdRR was higher compared to dogs (50) and cows (17). The pNN50 is the percentage of adjacent normal RR intervals that differ from each other by more than 50 ms and is a measure correlated with parasympathetic nervous system activity, also reflected by RMSS parameter (39). Our results were slightly higher compared to dogs (50), but similar to horses (49); considering the RMSS parameter, we found slightly higher values for dogs (50) and pigs (MF), and slightly lower for horses (49). Finally, the LF/HF value obtained in sheep was slightly lower than those of dogs (50), horses (49), and cows (22). The differences reported above may be due to several reasons: first, it is difficult to compare HRV biometric parameters between different species because they may be characterized by different autonomic nervous system activity and different behavioral responses to humans (17). Comparison between sheep populations might be useful, however, literature especially focused on HRV monitoring in ovine fetus for human medicine purposes (24–26). Moreover, HRV evaluation might change dramatically due to the circumstances of the measurements, including the device used for the monitoring and the length of monitoring (long-term monitoring vs. short- or ultra-short-term monitoring) (51).

Regression analysis of HRV parameters showed 4 out of 8 parameters with an R^2^ lower than 0.75. However, regression analysis is not the gold standard method for HRV estimation in ultra-short-term studies. Only one study (52) defined the minimal requirement for satisfactory concurrent validity (e.g., r = 0.9) for ultra-short-term measures. Thus, the literature recommended the use of Bland-Altman Limits of Agreement (39). The Bland Altman plots confirmed a general agreement between the two systems (Figures 4–11). The limits of the agreement were computed as Interquartile Range (IQR) using non-parametric statistics since the results of the Kolmogorov-Smirnov normality test applied to the distribution of the difference signal (53). Further, the recommendation of the International Organization for Standardization (54) about the expression of reliability in terms of standard deviations, the reproducibility coefficient (RPCnp) was calculated as 1.45 times the IQR of the differences between the two samples. The greater the value for RPCnp the less similar are the systems. Since RPCnp is very small almost for all extracted indexes, it indicates that Smartex and Televet systems are close to each other assuring an excellent accuracy of obtained results (55, 56).

The high coefficient of variation of LH/HF (reported in Table 2) showed a greater heterogeneity in the results. This could be due to a greater variation in the existing sympathovagal balance index between the sympathetic and parasympathetic modulation of the heart.

Different devices for HRV evaluation have been validated in different species. The Polar™ is one of the most common technologies used in animals for assessing HRV in cows (57), pigs (48), horses (49), and dogs (50). The authors evaluated the correlation and the agreement between the instruments using the same parameters provided in the present study (mean RR, pNN50, RMSSD, LF/HF, SampEn, SD1, SD2, stdRR), founding a higher correlation and agreement between the Polar^®^ device and the traditional ECG, as our study. Different results were found in cats in which Polar^®^ and ECG showed insufficient agreement (58).

Overall, according to the results of the present study, smart textiles can be considered as a reliable method to measure HRV in sheep, as already demonstrated in other species (59). However, some limits need to be addressed, such as the restricted number of sheep included, the enrolling of sheep from a single breed, and the performance of only “standing” monitoring for a relatively short time. The breed of the sheep might influence the length of the wool and the potential interference with HRV evaluation by artifacts. In our opinion, our results showing that HRV monitoring with Smartex technology is possible in breeds with short wool are promising for the field. Nevertheless, future research will need to include several sheep breeds with different wool characteristics and lengths in order to evaluate the need for shaving. Further studies will evaluate the Smartex technology while the animals are grazing and will include a wider population.

In conclusion, our study showed that the Smartex technology can be used for HRV evaluation in sheep species as a potential indicator for animal welfare assessment.

## Data availability statement

The raw data supporting the conclusions of this article will be made available by the authors, without undue reservation.

## Ethics statement

The animal study was reviewed and approved by the Institutional Animal Care and Use Committee of the University of Pisa (49/2019). Written informed consent was obtained from the owners for the participation of their animals in this study.

## Author contributions

LT: conceptualization, methodology, and writing–original draft preparation. FB: methodology, data curation, and writing–review and editing. AL: software, formal analysis, data curation, and writing–reviewing and editing. VV and IN: resources and data curation. MS: writing–reviewing and editing. MM: supervision, project administration, and writing–review and editing. All authors contributed to the article and approved the submitted version.

## Conflict of interest

The authors declare that the research was conducted in the absence of any commercial or financial relationships that could be construed as a potential conflict of interest.

## Publisher's note

All claims expressed in this article are solely those of the authors and do not necessarily represent those of their affiliated organizations, or those of the publisher, the editors and the reviewers. Any product that may be evaluated in this article, or claim that may be made by its manufacturer, is not guaranteed or endorsed by the publisher.
